# Comparing the Expression of Genes Related to Serotonin (5-HT) in C57BL/6J Mice and Humans Based on Data Available at the Allen Mouse Brain Atlas and Allen Human Brain Atlas

**DOI:** 10.1155/2017/7138926

**Published:** 2017-05-23

**Authors:** C. A. Acevedo-Triana, L. A. León, F. P. Cardenas

**Affiliations:** ^1^School of Psychology, Universidad Pedagógica y Tecnológica de Colombia, Tunja, Colombia; ^2^Universidad Sergio Arboleda, Bogotá, Colombia; ^3^Laboratorio de Neurociencia y Comportamiento, Universidad de los Andes, Bogotá, Colombia

## Abstract

Brain atlases are tools based on comprehensive studies used to locate biological characteristics (structures, connections, proteins, and gene expression) in different regions of the brain. These atlases have been disseminated to the point where tools have been created to store, manage, and share the information they contain. This study used the data published by the Allen Mouse Brain Atlas (2004) for mice (C57BL/6J) and Allen Human Brain Atlas (2010) for humans (6 donors) to compare the expression of serotonin-related genes. Genes of interest were searched for manually in each case (in situ* hybridization* for mice and microarrays for humans), normalized expression data (*z*-scores) were extracted, and the results were graphed. Despite the differences in methodology, quantification, and subjects used in the process, a high degree of similarity was found between expression data. Here we compare expression in a way that allows the use of translational research methods to infer and validate knowledge. This type of study allows part of the relationship between structures and functions to be identified, by examining expression patterns and comparing levels of expression in different states, anatomical correlations, and phenotypes between different species. The study concludes by discussing the importance of knowing, managing, and disseminating comprehensive, open-access studies in neuroscience.

## 1. Introduction

Since neuron theory was formulated, based to a large degree on the histological studies of Santiago Ramón and Cajal, there has been an awareness of the limitations of some experimental methods due to their simplicity and lack of detail, which fail to comprehensively address organisms, and of course a limitation in the explanation of phenomena from a cellular or anatomical perspective. This has become a constant criticism, despite this epistemological approach being repeatedly used in psychobiology for various practical, economic, and ethical reasons. One of the barriers to a comprehensive approach that incorporates various perspectives is the difficulty in bringing together a range of measures simultaneously. Thus, the effort to create brain maps or atlases represents a key tool for improving the validity of less comprehensive models [1–4]. Currently, the development of these techniques has led to hybrid biological and technical studies in both normal brains and brains that have been biochemically modified, to facilitate the study of their structure and functioning.

## 2. Genetic Atlases

An important aspect in neurobiological or anatomical approaches is to determine the common gene expression between the brain and other organs. Additionally, it has been found that not all brain structures present the same gene expression (some express particular genes to different degrees), but a large percentage of the entire genome is expressed at brain level [2, 5–7]. Many of these efforts to identify a pattern of gene expression are maps or atlases that allow us to “navigate” through the structures by following an expression pattern or locating specific points of interest [8]. These maps are now complemented by imaging techniques or computer modeling that allow them to be visualized and provide a global view of complete genomic expression [3, 4, 9–11].

Gene expression studies have become useful tools for understanding molecular aspects of psychobiological phenomena. They have helped refine anatomical [4, 9, 11, 14, 15], taxonomical [9, 16, 17], and statistical methods [18], which allow us to partly identify the relationship between structures and functions upon examining expression patterns [4, 14, 15, 19] and compare expression levels in different states [20] and anatomical correlations and phenotypes between different species [5, 14, 15, 18, 19].

Among the factors that have contributed to the dissemination and use of genetic databases is the availability of tools developed by the Allen Brain Atlas datasets. The Institute has made an atlas of genome expression for the mouse C57BL/6J and of the human genome publicly available, as part of the Allen Brain Atlas project [4, 6, 21].

A wide range of informative tools have been developed by the Allen Brain Atlas [4, 6, 22, 23]. The Allen Mouse Brain Atlas [24] includes an interactive reference that separates brain structures and simultaneously presents a collection of images with the in situ* hybridization* expression of each gene. Additionally, an open-access software known as* Brain Explorer*® [6, 14, 15, 18, 23] has been developed. This software allows a 3D visualization of the expression of more than 21,000 genes in brain structures.

Among the most interesting findings in the identification of genes is the subdivision in hippocampus structures. Traditionally, it has been believed that the hippocampus is divided into the dentate gyrus (DG) and* Cornu Ammonis* (CA1, CA2, and CA3), but, due to the gene expression in CA1, CA2, and CA3, it has been proposed that the cytoarchitectonic distribution does not suggest differences between these structures. An expression pattern of Prox1 (Prospero-related Homeobox 1) has been found in DG, Ptpru (Protein Phosphatase, receptor type, U) in CA1, Cacng (members of subunits of voltage-dependent calcium channels) in CA2, and Prss35 (protease, serine, 35) in CA3. Genetic mapping studies using ISH have also corroborated that the expression of similar genetic patterns of diverse areas presents similar functions [21].

As well as leading to the anatomical reclassification of structures based on expression, these data have allowed the distribution of specific protein expressions to be explored [25].

With respect to humans, The Allen Human Brain Atlas [26] was used for comparing expression in donors. Some studies have revealed that a high percentage of all genes (around 90%) are expressed in the brain; likewise, the cerebral cortex has a homogeneous molecular profile, with wider expression in the frontal lobes [11].

## 3. Serotonin (5-HT)

Serotonin or 5-hydroxytryptamine (5-HT) was chosen as the focus of this study for various reasons: first, because many behavioral disorders and the drugs used to treat them have been linked to serotonergic mechanisms [27, 38–40]; second, 5-HT projections and receptors have been described in multiple sites [14–16, 27], facilitating the search for the expression of related genes. Given that a broad area of research in psychobiology and behavioral neuroscience is the use of animal models, it is of interest to compare the distribution of serotonin-related gene expression, as this may help confirm the validity of interspecies models, in this case mouse-human [17, 41].

5-HT is related to physiological functions at peripheral (muscular, intestinal, and heart rate) and central level. Changes in its synthesis, bioavailability, and expression of receptors and other molecules related to its functioning have been associated with changes in behavior [12, 17, 27]. Many clinical conditions have been linked to altered serotonergic functions, including eating disorders, anxiety, issues with endocrine and circadian rhythm regulation, perception problems, compulsive behavior, pain, schizophrenia, sexual dysfunction, sleep disorders, and depression [12, 16, 27, 42, 43].

5-HT is synthesized from tryptophan obtained from dietary sources, primarily proteins and carbohydrates. A close relationship has been found between the quantity of tryptophan in a diet and 5-HT levels in the brain and mood [12, 44].

**Figure 1:**
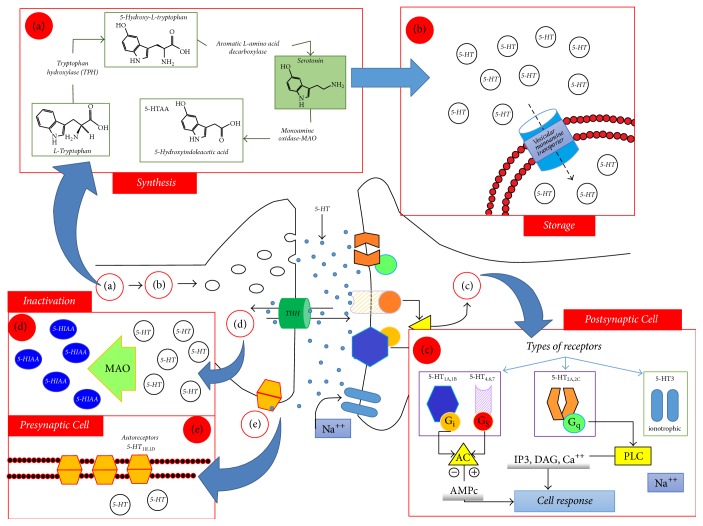
Representation of serotonergic synapses (synthesis, storage, liberation, and recapture). Processes related to serotonergic synapses. (a) Synthesis and metabolism: tryptophan is taken by the neuron through active transport mechanisms; (b) storage: once synthesized, it is stored in synaptic vesicles by vesicular transporters of monoamines (Vmat). When it is liberated, the serotonin interacts with various types of receptors; (c) postsynaptic receptors: the vast majority of postsynaptic receptors are G-protein coupled metabotropic receptors (HTR1A and HTR1B coupled to protein Gi and receptors HTR4, HTR6, and HTR7 are coupled to protein Gs) that activate adenylyl cyclase (AC), which in turn activates cyclic AMP, generating a cellular response; HTR2A and HTR2C are coupled to protein Gq and when activated, activate phospholipase C that triggers activity in inositol trisphosphate (IP_3_), diglyceride (DAG), and an increase in levels of intracellular calcium (Ca^++^), leading to a cellular response. The HTR3 receptors are postsynaptic and ionotropic, activated by ligands, allowing the flow of sodium (Na^+^) ions; (d) inactivation: once recaptured, the serotonin is inactivated by monoamine oxidase (MAO) enzymes, generating 5-hydroxyindoleacetic acid (5-HIAA); (e) presynaptic stimulation: HTR1B and HTR1D receptors modulate the liberation of serotonin. Adapted from [12].

The synthesis of 5-HT begins with tryptophan (Figure 1) which is hydroxylated to 5-HTP by the tryptophan hydroxylase enzyme (EC 1.14.16.4 controlled by the genes* TPH1* and* TPH2*). Only* TPH2* is expressed in the brain, and alteration on its expression is related to mental illnesses and risk of suicide [12, 32, 33]. Once 5-HTP is formed, it is decarboxylated by the aromatic L-amino acid decarboxylase (EC 4.1.128) [12]. This enzyme is controlled by the aromatic L-amino acid decarboxylase (AADC) gene [28, 45]. At this point 5-HT is produced. 5-HT can be catalyzed by monoamine oxidase (MAO) and aldehyde dehydrogenase (Figure 1) [12, 43]. After it is synthesized, stored, and released, reuptake of 5-HT is made by transporters and through this mechanism its action diminishes and synaptic homeostasis is maintained.

**Figure 2:**
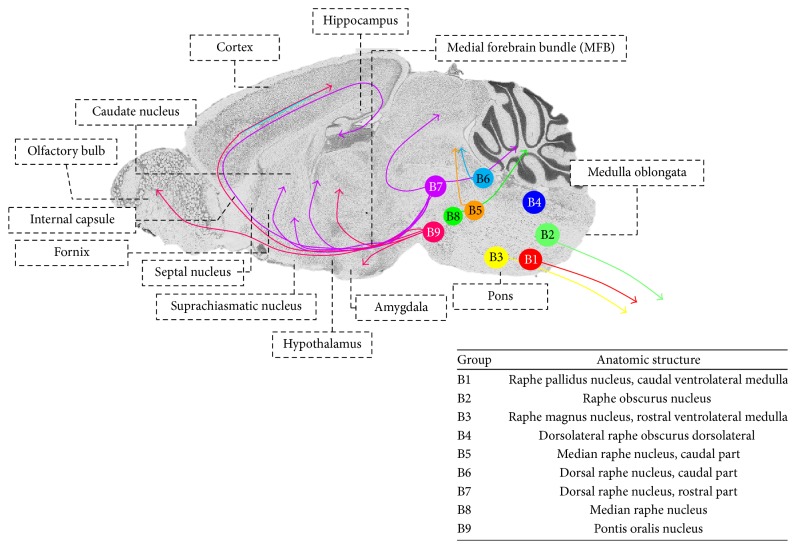
Location of serotonergic nuclei. Adapted from [12, 13].

Traditionally, it has been stated that the neurons that synthesize 5-HT are located in groups of cells in the midline and in the raphe nuclei of the pons and upper part of the brain stem (Figure 2). Nine serotonergic nuclei (B1–B9) have been described [12]. The caudal groups (B1–B3) project to the spinal cord, while the more rostral groups (B7–B9) project to the diencephalon and telencephalon, via two types of axonal terminals (thin and thick) that begin at the dorsal raphe nucleus and median raphe nucleus [12, 46, 47].

At least seven different types of receptors have been reported for 5-HT, some with receptor subtypes (5-HT_1_ [5-HT_1A_, 5-HT_1B_, 5-HT_1D*α*_, 5-HT_1D*β*_, 5-HT_1F_, 5-HT_1P_, and 5-HT_1S_]; 5-HT_2_ [5-HT_2A_, 5-HT_2B_, and 5-HT_2C_]; 5-HT_3_; 5-HT_4_; 5-HT_5_ [5-HT_5A_, 5-HT_5B_]; 5-HT_6_; 5-HT_7_). The location of serotonergic receptors can be seen in Figure 2. The majority of these are coupled to G-proteins except for 5-HT_3_ receptors that are coupled to an ion channel [12, 17, 48].

Several studies that have examined the functionality of receptor-related genes have shown that there are various polymorphisms of the HTR1A and HTR1B genes, genes related to the functionality of the hypothalamic-pituitary-adrenal axis and different responses to stress, suggesting particular forms of interaction [42, 49–51]. Likewise, alterations have been reported in the pattern of expression for the HTR1A and HTR2A genes in mental illnesses related to mood and response to stress [51, 52], panic attacks, [53], bipolar disorder, anxiety, and depression, among others [54–56], but not in behaviors such as suicide [49]. Reports of expression of the 5-HT1A receptor in the hippocampus, cortex, and hypothalamic regions correspond to the expression reported in this study [16, 55]. Other sources that provide data on serotonin genes are* postmortem* studies that report increases in genes such as HTR2A in patients with conditions such as depression and schizophrenia [54, 57, 58]. In the case of animal models, it has been reported that HTR1A knockout mice show behavioral responses of anxiety and depression [57].

The 5HT1F receptor participates in the effect of antimigraine drugs and is expressed in sensory neurons in the brain stem [59, 60]. With regard to the HTR1D gene, low expression levels have been reported in the basal ganglia, hippocampus, and cortex with involvement in phenomena of nociception and inflammation [59]. The affinity of certain antidepressive drugs with the 5HT1D receptor has been linked to phenomena of depression [59, 60].

With regard to the HTR1E gene, there are no studies that conclusively report on their function, but they are presumed to play a role in regulating emotions, anxiety, and attention-deficit disorders, hyperactivity, and Alzheimer's disease [59, 60]. As for 5HT2B receptors, antagonist drugs have been reported to seemingly induce hyperphagia and reduced self-care and social behavior [59, 60], Tourette syndrome [61], and some personality disorders [62].

The HTR2C gene has been implicated in various neurodevelopmental disorders [59, 60]. In fact, there are some reports on HTR2C polymorphisms related to autism [63], schizophrenia [59], mood disorders [64, 65], eating disorders [66], and some personality traits [67].

There is evidence of a link between the 5-HT3 receptor and thermoregulation processes [35, 68]. 5-HT3 receptor is expressed in regions related to ingesting food, processing pain, reinforcement systems, alcoholism, and cognition [69, 70]. It is interesting that not all the constituting subunits of the 5-HT3 receptor are expressed in the same way, but rather there is a high variability among them, suggesting that 5-HT3 receptors are structurally and functionally different due to differences in the subunits they consist of [71].

Expression of the HTR4 gene has been linked to drug-using behavior due to the high expression reported in reinforcement system structures [59]. For example, in this study, we observed the expression of this gene particularly in the striatal nuclei. Expression of the HTR5 and HTR6 receptor genes has been reported as occurring mainly in the hippocampus, cortex, basal ganglia, and cerebellum, and they have been connected with mood disorders, cognitive disorders, and schizophrenia [59]. In the data analyzed here we did not observe a particularly high expression of HTR5 or HTR6 in these structures. The HTR7 gene has been linked to physiological functions such as circadian rhythms, cognition, and mood [27, 72].

From plants to humans, these serotonergic mechanisms have been conserved [28]. Given the comparative approach of this study, we chose to compare the expression of genes related to the synthesis, storage, and release of 5-HT both in mice (C57BL/6J) and in microarray studies of humans, using the data reported by the* Allen Institute for Brain Science* (http://www.brain-map.org) as a source. Although the comparison is on quantitative data it is only possible to make a qualitative comparison and explore visual relations and validation in methods for later studies.

## 4. Methods

Here we used an exploratory design in databases using a web-based application for visualizing expression energy of genes called Allen Brain Atlas-Driven Visualizations (ABADV) (http://www.socsci.uci.edu/~jkrichma/ABADV/) [73]. Atlas reference tools (for both mice and humans), images of expression levels,* Brain Explorer *for the visualization and presentation of data, and the AGEA tool to relate expression levels in different areas were also used (procedure details for databases in Table S1, in Supplementary Material available online at https://doi.org/10.1155/2017/7138926).

Specifically, ABADV works by extracting quantified gene expression energy values per 200 *μ*m voxel of the mouse brain. The expression energy is defined as the sum of expression pixel intensity for each gene divided by the sum of all pixels in division. The energy expression (*E*) for each gen is represented in a voxel where “weighted sum of the greyscale-value intensities *I* evaluated at the pixels *p* intersecting the voxel

(1) $$\begin{array}{c} E\left(\;v,g\;\right)=\frac{\sum_{p\in v}M\left(p\right)I\left(p\right)}{\sum_{p\in v}1}, \end{array}$$

where *v* is voxel, *g* is gene, *M*(*p*) is a Boolean mask that equals 1 if the gene is expressed at pixel *p* and 0 if it is not” [74, p 7].

Expression energy is then computed for each brain structure delineated in the Allen Mouse Brain Atlas [24] and Allen Human Brain Atlas [26]. This study took the data reported in database independently for mice (C57BL/6J) and for humans.

For ISH the patterns of gene expression in mice were obtained in studies of 8-week old male C57BL/6J mice, using a semiautomated process in which the brain was divided into 25 *μ*m sections at intervals of 100 *μ*m to 200 *μ*m [18, 21]. The slices were then hybridized with digoxigenin- (DIG-) labeled riboprobes. Finally, information was captured using a camera with 0,95 *μ*m/pixel resolution and then analyzed and quantified using software for measuring signal intensity [18]. Despite the limitations of this method for identifying expression, it can be assumed to be reliable thanks to the control procedures and similar corroboration techniques [18].

Table 1 lists the genes that were searched for analysis.

For human data, the expressions of genes were download of the complete dataset of six human donors from the Allen Human Brain Atlas (http://human.brain-map.org/). We analyzed human gene expression data, obtained using the microarray technique, through a histological analysis and a microarray profile of more than 900 structures in two individuals initially [11]. The procedure implies that they collected “approximately 500 anatomically discrete samples from cortex, subcortex, cerebellum, and brainstem of each brain and profiled for genome-wide gene expression using a custom Agilent 8 × 60K cDNA array chip” [3]. More information about collect and extraction information can be found in http://help.brain-map.org/display/humanbrain/documentation/. Each gene of Table 1 was searched for and download in R, after a matrix with values was done. The missing values (NAs) were removed and a heatmap with dendrograms was made for each donor (Figure 4).

The data of each of the donors was provided by Allen Human Brain Atlas [26]. Metadata associated with donor attributes such as age and postmortem interval (PMI) are described (Allen Human Brain Atlas, Case Qualification and Donor Profiles: http://help.brain-map.org/download/attachments/2818165/CaseQual_and_DonorProfiles.pdf?version=1&modificationDate=1382051848013) and summarized in Table 2.

All data for this part of study were downloaded from Allen Human Brain Atlas [26] (Table S1) and were preprocessed by software packages included in the R-project (https://www.r-project.org/) or Bioconductor (http://www.bioconductor.org). An unsupervised hierarchical clustering analysis was made of the rows of each matrix data. So, the data matrix is reordered according to the hierarchical clustering result, putting similar observations close to each other. The blocks of “high” and “low” expression are adjacent in the data matrix. The method of measuring distances was Euclidean. Before cluster analysis, the matrix data were standardized using the function scale⁡(), and other packages used were cluster, factoextra, and gplot.

## 5. Procedure

**Figure 3:**
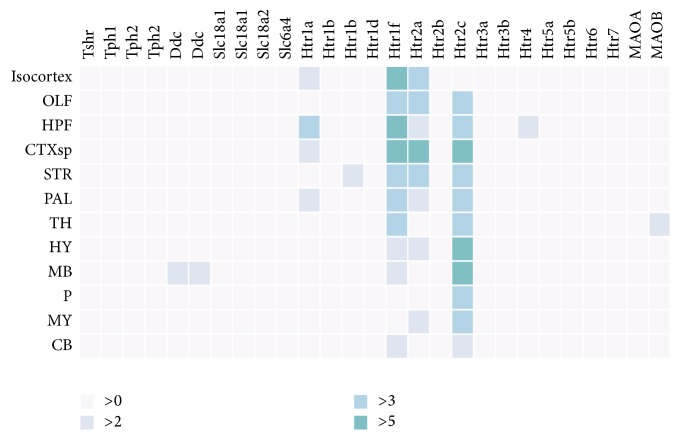
Expression of serotonin genes in each of the brain structures of the C57BL/6J mouse using ABADV.

In the case of mice (C57BL/6J), we opened the Allen Mouse Brain Atlas [24] (http://mouse.brain-map.org) and, under the “Mouse Brain” option in search box, we manually entered each of the genes related to 5-HT (Table 1) within the “Gene Search” option. We considered data from coronal and sagittal sectioning. Of ABA the name was extracted. After that, we accessed ABADV (http://www.socsci.uci.edu/~jkrichma/ABADV/) and in genes box we put these search terms: “Tshr, Tph1, Tph2, Ddc, Slc18a1, Slc18a2, Slc6a4, Htr1a, Htr1b, Htr1d, Htr1f, Htr2a, Htr2b, Htr2c, Htr3a, Htr3b, Htr4, Htr5a, Htr5b, Htr6, Htr7, Maoa, Maob” and in Brain structure box we put “Isocortex, OLF, HPF, CTXsp, STR, PAL, TH, HY, MB, P, MY, CB” (Figure 3).

In the case of human data, we entered the main brain atlas page and selected the “Human Brain” option. We selected “Microarrays” and entered each of the genes listed in Table 1 and then downloaded the data associated with gene expression for each one from the corresponding visualizations on the page. Unlike the data for mice, expression data for humans is divided into more than 1000 structures for 6 donors.

Levels of expression were taken as similar values as ISH scales (from 0 to 10 in *z*-scores) while in microarrays the scale ranges from negative values (approximately −5) to positive (approximately +5). A reading of over 5 was assumed to be high for mice and over 2 for microarrays in the case of humans.

## 6. Results

### 6.1. C57BL/6J Mice: In Situ Hybridization

Figure 3 presents the energy expression of all genes in each structure, allowing a visualization of the greatest quantities of energy expression (EE) in each brain structure. The results were analyzed with the following scale: >5 (high energy expression), >3 (medium energy expression), >2 (moderate energy expression) and >0 (low energy expression) according to heatmap from ABADV results. This scale only was applied in heatmaps. In general terms the genes with major EE were receptors genes (Htr1f, Htr2c, Htr1f, and Htr1a) at different structures. It is important to indicate that the general EE is low due to comparing all genes at the same time.

As expected, greater expression of the genes that are involved in the synthesis and storage of 5-HT was observed in the midbrain, hypothalamus (with DDC and Scl18a2 [VMAT-2]), and the pons, medulla and cerebellum with a significant presence of VMAT-2 and TPH-2 genes, also related to the synthesis and storage of 5-HT (Figure supplementary S1).

A marked expression of genes related to the 5-HT1F and 5-HT2A receptors is present in forebrain structures, with lower expression in midbrain structures. There was found a great expression of genes HTR1F, HTR2A, and HTR1A in the isocortex. These receptors are also expressed in the olfactory bulb, but there is a marked expression of the gene for the 5HT2C (HTR2C) receptor in more posterior structures, reaching its highest level in the midbrain. With respect to other receptors, these were generally low in EE, with the HTR3B gene pronounced in the olfactory bulb, hypothalamus, globus pallidus, hippocampal formation, and medulla. Medium expressions of the HTR4 receptor and HTR1A were also found in the hippocampal formation (Figure supplementary S2).

Finally, the genes related to the inactivation of 5-HT present low expression of the gene that codes for the expression of MAOA is seen in all structures, while the gene that codes for the monoamine oxidase enzyme MAOB has medium expression in the thalamus and low expression in the hypothalamus, midbrain, and pons (Figure 2 Supplementary).

### 6.2. Humans: Microarrays

It was possible to download microarray data on human gene expression directly from the Allen Human Brain Atlas [26] (http://human.brain-map.org). To obtain expression levels we used the same procedure for mice data in terms of searching for each particular gene. Once the results were shown, we downloaded them and grouped related genes. Expression data for each human gene contained more than 1000 data points in different brain structures for donors.

The normalized expression values (*z*-scores) for each of the genes in the different brain structures for brain donors were put in Table 1 Supplementary. It is worth highlighting that these expression values were represented across a color scale from green to red, where dark green represents low expression levels and red high expression levels. For this analysis, the expression value reported at the website was used. Thus, negative values indicate low levels of expression, and positive values, high levels.

**Figure 4:**
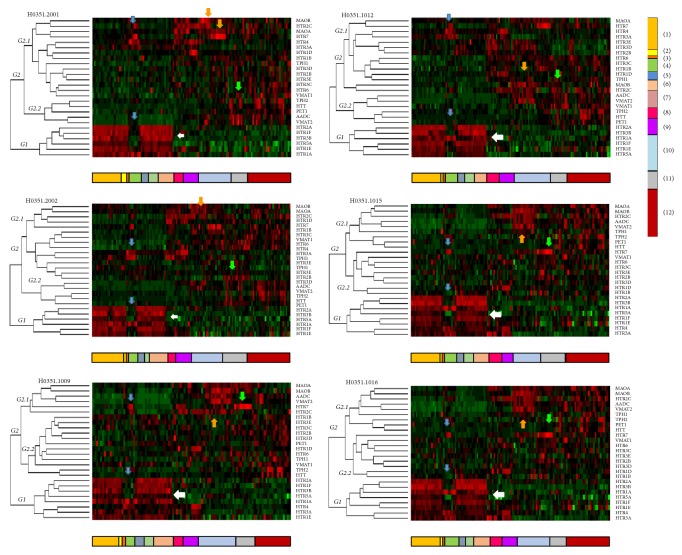
Heatmaps of gene expression of serotonin system in donors. On the left, dendrogram with organization in two main groups (G1 and G2). The second group has two branches or trees. Below each heatmap a color representation of brain structures. The original heatmap for each donor in supplementary images. Group 1 is related to height expression in structures at cerebral cortex in general (white arrow). The hippocampal formation and together structures (dentate gyrus, CA1, CA2, and CA3) present low expression to compare with cortex but height expression in HTR1A gen and HTR4 and HTR7 (blue arrow). The orange arrow shows a group of genes related to inactivation MAOA, for example, and green arrow shows genes related to synthesis and storage. The column on the right has the convention of colors: (1) Frontal Lobe; (2) Insula; (3) Limbic Lobe; (4) Hippocampal Formation; (5) Occipital Lobe; (6) Parietal Lobe; (7) Temporal Lobe; (8) Amygdala; (9) Basal Ganglia; (10) Diencephalon; (11) Mesencephalon; (12) Hindbrain.

Viewing the results together in Figure 4 after to make the heatmaps several peculiarities of the gene expression was observed. The division groups in dendogram are mainly two. In the first group is a high expression in the cortex structures generally several receptor genes, showing high expression levels. This expression occurs in 6 donors similarly (Figure 4, white arrow). In the first group also appear low expression of genes regarding hippocampal structures, where, clearly there is a low gene expression in cortex in general and high expression of receptors HTR4 and HTR7 which could be a specific marker of structures (Figure 4, blue arrow). In the second group of divisions or dendrogram are grouped depending on the function of genes. So that genes for the synthesis and storage have expression in structures of mesencephalon while genes that are related wiht inactivation are expressed in the diencephalon in general. Another group that appears is related to genes generally have low expression at brain and mostly are receptor genes. There are differences between donors but are obvious similarities in the formation of groups and expression in terms of functions and processes related to specific structures within the serotonergic system.

Figure 4 shows the expression of genes related to the serotonin system in humans. In general, high expression of all genes related to synthesis and storage (orange arrow) can be observed in the human midbrain, in a similar way to that which occurs in mice. In the striatum and hypothalamus, high expression of the gene that codes for AADC enzyme can be seen. In the hypothalamus there is also high expression of the gene for the vesicular transporter VMAT2. High expression of these synthesis and storage genes is also present in the brain stem and hindbrain. There are medium/low and isolated expression in the amygdala (TPH-1 and VMAT-1), globus pallidus (AADC) and hippocampal formation (TPH-2 and VMAT-2). It was found that when the variant TPH-1 is expressed, VMAT-1 tends to be expressed as well, and when THP-2 is expressed, so is VMAT-2.

In relation to genes that code for receptors, there is medium expression of the HTR1A gene in the frontal lobe, and high expression in the insula, cingulate gyrus, claustrum and hippocampal formation in donors. Likewise, low expression of HTR1A can be observed in the parietal lobe, amygdala, basal ganglia, diencephalon, midbrain, brain stem and white matter. The HRT1D gene has low expression in most structures and high expression in the striatal nuclei while the HTR1E gene has high expression in forebrain structures (frontal lobe, insula, cingulate gyrus, parietal lobe, basal forebrain) and very low expression in the epithalamus, hypothalamus, thalamus, midbrain and brain stem. Similar expressions are found for HTR1F and HTR2A. It is interesting to observe that the expression is low in the hippocampal formation. The HTR2C receptor gene is low in the structures of the cortex, increasing in the basal ganglia and decreasing in the brain stem while HTR2B is particularly medium in the midbrain.

The HTR3A gene is expressed in various structures in the hippocampus (blue arrow) and amygdala, with lower expression in diencephalic structures and brain stem. The gene for the HTR3B receptor appears to be expressed more in areas of the brain cortex and less in subcortical structures. Expression for the 5HT5A receptor gene (HTR5A) follows a similar pattern. Expression levels for HTR3C are low at the cortical level and medium in midbrain and brain stem. The same patterns is found for HTR6. As for HTR3D and HTR3E genes, they have low levels in the amygdala and midbrain, brain stem and myelencephalon. The expression of HTR4 is pronounced in the basal ganglia. The gene of the HTR7 receptor has relatively high expression in the thalamus, as opposed to other structures.

Genes related to the inactivation of serotonin have low expression in forebrain structures but its expression increases in the amygdala, basal forebrain, globus pallidus and striatum. Expression of MAOB is also particularly high in the epithalamus and hypothalamus, but low in the cerebellum and white matter. For mice, high levels of gene expression for MAOB occur only in the thalamus (orange arrow).

### 6.3. C57BL/6J-Human Comparison

**Figure 5:**
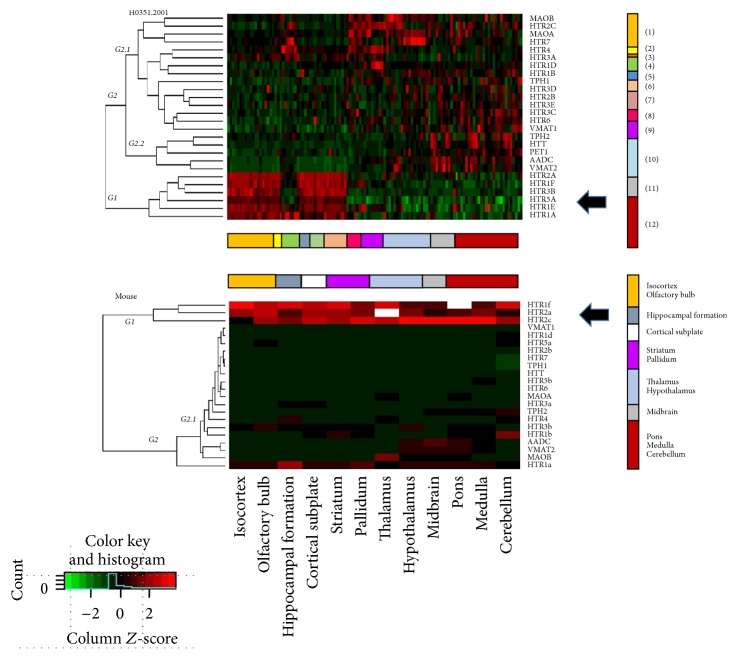
Heatmaps of gene expression of serotonin system in donor H0351,2001 (microarray) and mouse (hybridisation in situ). On the left, dendrogram with organization in two main groups (G1 and G2). The second group has two branches or trees. Below each heatmap a color representation of brain structures. Group 1 is related to height expression in structures at cerebral cortex in general (white black) in both species. The column on the right for donor has the convention of colors: (1) Frontal Lobe; (2) Insula; (3) Limbic Lobe; (4) Hippocampal Formation; (5) Occipital Lobe; (6) Parietal Lobe; (7) Temporal Lobe; (8) Amygdala; (9) Basal Ganglia; (10) Diencephalon; (11) Mesencephalon; (12) Hindbrain. Convention in mouse expression was made similar to donor.

In Figure 5 heatmaps of gene expression of serotonin system in donor H0351,2001 (representatively of donors) (microarray) and Mouse (Hibrydation In Situ) are qualitative compared. It is important to signal that in cortex the expression genes are similar suggesting comparative system in spite of differences in methods in expression.

Genes involved in the synthesis of serotonin display a very similar expression pattern in ISH and microarray data: Low levels are present in cortical structures, hippocampus and basal ganglia, while higher levels are present in the midbrain and posterior structures (pons, brain stem and hypothalamus). It should be noted that expression in the hypothalamus and midbrain is almost identical between the two species with respect to ADDC, HTT and VMAT2 genes. In mice was difficult to find exactly the same genes as in humans because these genes had no data available in database, so we only could to compare one part.

The HTR1A, HTR2A, and HTR1F genes are highly expressed in all areas of C57BL/6J mice, but in humans these only appear to be highly expressed in some areas of the cortex (frontal lobe, parietal, and cingulate gyrus) and hippocampal formation, with a difference in terms of subcortical structures: while expression in humans is not high, in C57BL/6J this expression is maintained. It is particularly interesting that the HTR1F gene displays highest levels of expression in the entire brain for mice and in the brain cortex for humans. The HTR1E gene is only expressed in humans. When we compared 5-HT1A, main presynaptic regulator of 5-HT, we found that in humans was highly expressed in cortex and lowly in other areas, conversely in mice the expression profile is similar between areas and in dendrogram was separated as only one but been similar in isocortex.

With regard to the HTR1B gene, it has low expression in mice and middle/low expression in humans, being almost equivalent to 0. Expression of HTR1D is low in nearly all zones but has high expression in the striatal nuclei in humans only. The HTR2B gene has low levels of expression in mice and medium expression in humans, with medium/high expression in the amygdala, forebrain, and structures close to the midbrain. The HTR2C gene has medium expression in all structures of mice, being greatest at the subcortical level and in the midbrain. For humans, its expression is low in some areas of the cortex and medium/low in the amygdala, basal forebrain, striatum, claustrum, epithalamus, hypothalamus, and midbrain.

In relation to genes that code for subunits of the HTR3 receptor, HTR3A and HTR3B have low expression in mice, with levels marginally higher for HTR3B. The profiles for HTR3A and HTR3B are not as low for humans; these are expressed more in anterior areas and less in posterior areas. In the case of genes for HTR3C, HTR3D, and HTR3E subunits, these are only reported in humans at relatively low levels. Genes HTR4, HTR5A and HTR5B in the case of mice, HTR6 and HTR7, have low levels of expression in all areas. The expression of HTR3 is similar between humans and mice but seems to be different for the other genes. HTR5A, for example, is expressed more in anterior areas and less in posterior areas, but with increased expression in the cerebellum. On the other hand, the level of expression for HTR6 is greater in posterior areas and lower in anterior areas. HTR7 expression varies between structures with no clear expression profile.

As for serotonin inactivation, expression of MAOA and MAOB is low in all mouse structures, with the exception of the thalamus, where expression of the gene for MAOB is high. This same pattern of expression is also found in the same structures in humans, with the only difference in the MAOA expression in the amygdala and basal ganglia and in MAOB expression in diencephalic and midbrain structures. This difference between 5-HT related genes expression and MAO gene related expression is probably owing to the diverse functions of MAO that are not only related to serotonin.

## 7. Discussion

The high levels of expression in genes related to synthesis and storage in areas close to the midbrain suggest that these areas may not be functionally separated; instead there seems to be an influence in expression according to the activity of each of these areas [4, 75, 76]. This idea could be corroborated using two tools also developed by the Anatomic Gene Expression Atlas-AGEA (http://www.brain-map.org/agea/) that shows a correlation in gene expression in nearby areas [4] as shown in Figure supplementary S9 and the expression of one connectivity marker injected in the dorsal raphe nucleus shown in Figure supplementary S10. These images could help to compare different topics for analysis available in Allen Brain Atlas datasets.

We found that qualitative comparisons in the expression of serotonin genes in brain in both species could be a new exploratory method for the validation of translational studies. Some studies in large scale have made comparisons of complete genomes or whole brain found specific biomarkers for each species [19, 77–80]. However, comparisons for particular systems (serotonin, e.g.) have not been extensively explored, wasting the potential of the so-called big data studies and that could have forward applications in health and translational studies and bettering the understanding in the relationship of the brain and behavior [81–83]. The results reported show functionally the relationship of serotonin gene expression with analogous structures between mice and humans. Some studies report similarities in this sense; however, these comparisons usually are in specific genes [84, 85]. In this study we report the comparisons in the complete genes related to serotonin.

The comparison technique proposed here suggests that the expressions of genes related to the synthesis, storage, reception and—atsome degree—inactivation of serotonin share similar mechanisms and locations between mice and humans. It is important to show that analysis between donors havs similar patterns of expression or group of genes suggesting a genetic mark or physiological phenotype such as has been proposed in similar studies [3]. These genetic similarities strengthen the validity of using animal models to study the functionality of genes and behaviors. It also enables the viability and functionality of some of these models and validates interspecies comparisons in physiology, genetics, and behavior. Accordingly, recent reports have attempted to identify percentages of similarity and difference between rodent and human cerebral cortex. These percentages of similarity and difference can then be taken into account when using behavioral and molecular models [80].

It is important to note that this study has major limitations that should be kept in mind when attempting to extrapolate the data or use the results as absolute measures. First, the data presented are produced from two techniques (in situ hybridization and microarrays) that use similar principles but different methods. Mouse data treats the principal areas of the brain as homogenous while for human data there is a great range of expression sites within each structure, and thus it is highly likely that averaging the expression data hides variations in expression levels.

Second, when comparing expression in human structures it is possible to better discriminate between areas because this comparison is more comprehensive in humans. This also makes interspecies comparison more difficult. Third, expression methods in mice relate to a large sample size (more than 1000 animals) while for humans they relate to six donors. It must be mentioned that although the Allen Human Brain Atlas presents data from six individuals, the expression levels between subjects were analyzed, and similarity was found in the majority of genes; qualitative comparison data was included in this study but quantitative comparison is not made.

Finally, the data was taken in a static way, without considering epigenetic measures, which, at least in the case of humans, could explain differences in expression [86, 87]. This may limit the generalization of the study to other humans. Despite these limitations, this study has various objectives beyond being exhaustive in terms of comparative biology. It aims to present high-tech, open-access tools that employ standardized methods, as well as showing their usefulness in interspecies comparisons, allowing an initial assessment in the use of translational studies between mice and humans.

With respect to the data from comprehensive neurobiological, genetic, or physiological studies, there is an increasingly greater effort to create a comparative view of different species that leads to interaction between groups and the undertaking of projects that specialize in macroscopic and microscopic aspects, as well as studies into levels of genetic or physiological function, providing as a result a wide range of tools and information repositories, some of them under open-access policies [10, 20, 88]. The efforts of the IABS, in this study, have brought about significant progress in four principal aims addressed by its projects [6, 23]: bioinformatics, management of large-scale information, data processing, and open-access data presentation [6, 14, 15].

Furthermore, the many efforts to develop open-access projects have created an enormous quantity of information that in many cases has led to networks forming for cooperation and checking of results. This is a way of validating the experiments that help improve and strengthen studies, efforts and discussion between groups [14, 15].  Table 3 lists some of these studies, databases, and tools in the field of neurobiology.

## Supplementary Material

Procedure details about databases, URLs and search.

## Figures and Tables

**Table 1:** List of genes to various serotonin-related processes considered in this study.

Process	Genes	Gene ID	Name	Function	References
Neurodevelopment	PET-1	260298	FEV (ETS oncogene family)	Development of serotonin neurons	[12, 27–30]

Enzymatic synthesis	TPH-1	21990	Tryptophan hydroxylase	Converts tryptophan in 5-hydroxytriptophane	[12, 27, 31]
	TPH-2	121278	Tryptophan hydroxylase	Converts tryptophan in 5- hydroxytryptophan (exclusive for brain tissue)	[12, 27, 31–33]
	AADC [nonspecific gen in 5-HT metabolism]	13195	aromatic enzyme decarboxylase	Converts 5-hydroxytryptófano into 5-HT and catalyzes different decarboxylation reactions with monoamines	[12, 27, 28]

Store	VMAT-1 [nonspecific gen in 5-HT metabolism]	110877	Monoamine vesicular transporter-1	Vesicular storing of 5-HT and transport other monoamine neurotransmitters	[12, 27, 28, 34]
	VMAT-2 [nonspecific gen in 5-HT metabolism]	25549	Monoamine vesicular transporter -2	Vesicular storing of 5-HT and transport other monoamine neurotransmitters	[12, 27, 28, 34]
	5HTT (Slc6a4)	15567	Serotonin transporter	5-HT reuptake	[12, 27, 28]

Receptor interaction	HTR1A	15550	Serotonin receptor 1A	5-HT Receptor coupled to a Gi-o protein (pre and postsynaptic)	[12, 27, 28, 35]
	HTR1B	15551	Serotonin receptor 1B	5-HT Receptor coupled to a Gi – protein (pre and postsynaptic)	[12, 27, 28, 35]
	HTR1D	15552	Serotonin receptor 1D	5-HT Receptor coupled to a Gi – protein (pre and postsynaptic)	[12, 27, 28, 35]
	HTR1F	15557	Serotonin receptor 1F	5-HT Receptor coupled to a Gi – protein	[12, 27, 28, 35]
	HTR2A	15558	Serotonin receptor 2A	5-HT Receptor coupled to a Gq	[12, 27, 28, 35]
	HTR2B	15559	Serotonin receptor 2B	5-HT Receptor coupled to a Gq	[12, 27, 28, 35]
	HTR2C	15560	Serotonin receptor 2C	5-HT Receptor coupled to a Gq	[12, 27, 28, 35]
	HTR3A	15561	Serotonin receptor 3A	Receptor coupled to an ion cannel	[12, 27, 28, 35]
	HTR3B	57014	Serotonin receptor 3B	Receptor coupled to an ion cannel	[12, 27, 28, 35]
	HTR4	15562	Serotonin receptor 4	5-HT Receptor coupled to a Gs	[12, 27, 28, 35]
	HTR5A	15563	Serotonin receptor 5A	Receptor coupled to a Gi and Go	[12, 27, 28]
	HTR5B	15564	Serotonin receptor 5B	Receptor coupled to a Gi and Go	[12, 27, 28, 35]
	HTR6	15565	Serotonin receptor 6	Receptor coupled to a GS	[12, 27, 28, 35]
	HTR7	15566	Serotonin receptor 7	Receptor coupled to a GS	[12, 27, 28, 35]

Degradation	MAOA [nonspecific in 5-HT metabolism]	17161	Monoamine oxidase-A	Inactivation of 5-HT and other monoamines	[12, 27, 28]
	MAOB [nonspecific in 5-HT metabolism]	109731	Monoamine oxidase-B	Inactivation of 5-HT and other monoamines	[12, 27, 28]

**Table 2:** Summary patient characteristics from Allen Human Brain Atlas [26] (http://human.brain-map.org).

Donor	Age (years)	Sex	Ethnicity	Postmortem interval (hours)
H0351.1009	57	M	White or Caucasian	26
H0351.1012	31	M	White or Caucasian	17
H0351.1015	49	F	Hispanic	30
H0351.1016	55	M	White or Caucasian	18
H0351.2001	24	M	Black of African American	23
H0351.2002	39	M	Black of African American	10

Details of qualitative and description of procedure and donors profile in http://help.brain-map.org/download/attachments/2818165/CaseQual_and_DonorProfiles.pdf?version=1&modificationDate=1382051848013.

**Table 3:** Internet databases and tools for exploring diverse neurobiological, genetic, cellular, and physiological data.

Resource (database-DB/Tool-T)	Web address	Purpose
Reference atlas	http://atlas.brain-map.org/	Interactive reference atlas for mice and humans

Anatomic Gene Expression Atlas – AGEA (DB/T)	http://www.brain-map.org/agea/	Atlas of genic expression that allows users to visualize the correlation of expression patterns between areas of interest

Brain Explorer 2 (H)	http://mouse.brain-map.org/static/brainexplorer	Open-access software for visualizing the structures and expression of all mouse, human, and neurodevelopment genes

Brain Architecture Knowledge Management System (BAMS) Ontological neuroanatomical (DB)	http://neuro.imm.dtu.dk/wiki/Brain_Architecture_Management_System	Development of a semantic framework for classifying types of neurons and classes

Cell Centered Database (CCDB) (DB)	http://ccdb.ucsd.edu/	Database of images in 2D, 3D, and 4D

Gene Expression Nervous System Atlas (GENSAT) (DB)	http://www.gensat.org/	Detection of the expression of genes in the mouse CNS with genetic techniques and with transgenic animals

GenePaint (DB)	http://www.genepaint.org/	Digital atlas of genetic expression patterns in adult mice and during development.

International Neuroinformatics Coordinating Facility (INCF) (DB)	http://www.genepaint.org/	Neuroinformatic tools for searching other databases and sources

NeuroMorpho (DB)	http://neuromorpho.org/	Digitally reconstructed neurons

NeuroGateway (DB)	http://neurogateway.org/catalog/goto.do?page=.sfngateway	Neuroscience sources, data and tools

Brain Architecture Management System (BAMS) (DB)	https://bams1.org/	Neuronal projections in the mouse brain

Collations of Connectivity Data on the Macaque Brain (CoCoMac) (BD)	http://cocomac.org	Neural projections in the macaque brain

Functional Anatomy of the Cerebro–Cerebellar System (FACCS) (DB)	http://www.rbwb.org/	3D atlas of axonal pathways in mice

BrainMaps.org (DB)	http://brainmaps.org	Interactive brain atlases for various species

Database on connectivity in humans (DB)	http://brainarchitecture.org	Postmortem study databases on connectivity in humans

Brain connectivity database (DB)	http://www.cma.mgh.harvard.edu/ibcd/	Database of studies on connectivity in humans.

Surface Management System DataBase (SumsDB) (DB)	http://brainvis.wustl.edu/wiki/index.php/Sums:About	Atlas showing connection density in macaques

SynapseWeb (BD)	http://synapses.clm.utexas.edu/	Reconstruction structures from electron microscopy images

Neocortical microcircuit database (DB)	https://bbp.epfl.ch/nmc-portal/welcome	Database of cellular connections

Atlas ICBM DTI-81 (DB)	http://www.loni.usc.edu/ICBM/Downloads/Downloads_DTI-81.shtml	Atlas of diffusion tensor imaging in humans

Anatomy Toolbox Fiber Tracts (DB)	http://www.fz-juelich.de/ime/spm_anatomy_toolbox	Atlas of white matter connectivity

WormAtlas (DB)	http://www.wormatlas.org	Atlas of C. elegans neurons

Molecular Anatomy of the Mouse Embryo Project (MAMEP) (DB)		Functional analysis of gene expression during development

EMBRYS (DB)	http://integbio.jp/dbcatalog/en/record/nbdc00867	Functional analysis of gene expression during development

EURExpress (DB)	http://www.eurexpress.org	Neuroanatomical atlas of transcriptome development in mice

The Brain Gene Expression Map BGEM (DB)	http://www.stjudebgem.org	Detect gene expression in the mouse CNS using genetic techniques and transgenic animals

Adapted from [9, 10, 36, 37].
