# Supplementary material for: Comparing the Expression of Genes Related to Serotonin (5-HT) in C57BL/6J Mice and Humans Based on Data Available at the Allen Mouse Brain Atlas and Allen Human Brain Atlas
Source: Neurol Res Int. 2017 May 23;2017:7138926. doi: 10.1155/2017/7138926 (PMC5463198; doi:10.1155/2017/7138926)
Supplement: Supplementary file 1 — Procedure details about databases, URLs and search. [file 7138926.f1.zip › Supplementary files/Table S1 suppl.docx]

| **Database** | **Permalink** | **Purpose** | **Procedure** | **Result** |
| --- | --- | --- | --- | --- |
| Allen Brain Atlas-Driven Visualizations (ABADV) | <http://www.socsci.uci.edu/~jkrichma/ABADV/> | To visualize expression energy of genes of mouse specifically serotonin system | We searched this genes: “TPH-1, TPH-2, AADC, VMAT-1, VMAT-2, HTT, HTR1a, HTR1b, HTR1d, HTR1f, HTR2a, HTR2b, HTR2c, HTR3a, HTR3b, HTR4, HTR5a, HTR5b, HTR6, HTR7, MAO-A, MAO-B” in this structures: “Isocortex, Olfatory bulb, Hippocampal formation, Cortical subplate, Striatum, Pallidum, Thalamus, Hypothalamus, Midbrain, Pons, Medulla, Cerebellum” | Heatmap with energy expression presented in Figure 3 |
| Brain Explorer © 2012 | <http://mouse.brain-map.org/static/brainexplorer> | Application developed by the Allen Institute for visualizing expression data. Can to display ISH expression data from multiple genes superimposed on each other in 3D | We searched each genes: “TPH-1, TPH-2, AADC, VMAT-1, VMAT-2, HTT, HTR1a, HTR1b, HTR1d, HTR1f, HTR2a, HTR2b, HTR2c, HTR3a, HTR3b, HTR4, HTR5a, HTR5b, HTR6, HTR7, MAO-A, MAO-B” images for representation and anatomical expression were made. We navigated through their structural ontology panel within the application. | Images are no show in text, procedures and purpose similar to reported by Lau et al., 2008 |
| Allen Mouse Brain Atlas (2004) | <http://mouse.brain-map.org> | To visualize expression energy of genes of mouse specifically serotonin system | We searched each genes in table 1. Only below genes have result with quality procedures: “TPH-1, TPH-2, AADC, VMAT-1, VMAT-2, HTT, HTR1a, HTR1b, HTR1d, HTR1f, HTR2a, HTR2b, HTR2c, HTR3a, HTR3b, HTR4, HTR5a, HTR5b, HTR6, HTR7, MAO-A, MAO-B”, the scores of energy expression were downloaded. This atlas represents quantified gene expression energy values per 200 μm voxel of the mouse brain. | The data were compared and compute through ABADV web application. |
| Allen Human Brain Atlas (2010). | <http://atlas.brain-map.org/> | To visualize expression energy of genes of humans specifically serotonin system in each donors | The expression of genes were download of the complete data set of six human donors. We searched each genes in table 1. A CVS file contain data for each gen in each donor. After a matrix with values was built. Analysis of clustering and dendrograms were made. | Figure 4, 5 and Figure S3 – S8 |
| Anatomic Gene Expression Atlas – AGEA (DB/T) | [www.brain-map.org/agea/](http://www.brain-map.org/agea/) | To compare expression correlated in areas in Allen Mouse Brain Atlas mainly nuclei serotoninergic (raphe dorsal, pons and brainstem). | We searched each genes: “TPH-1, TPH-2, AADC, VMAT-1, VMAT-2, HTT, HTR1a, HTR1b, HTR1d, HTR1f, HTR2a, HTR2b, HTR2c, HTR3a, HTR3b, HTR4, HTR5a, HTR5b, HTR6, HTR7, MAO-A, MAO-B” images for representation and anatomical expression were made. The main structures were associated with nuclei serotoninergic anatomically. | Comparisons for each gen, results showed in Figure S9. Details and technical procedures and purpose similar to reported by Ng et al., 2009 |
