# Supplementary figures and images for: Comparing the Expression of Genes Related to Serotonin (5-HT) in C57BL/6J Mice and Humans Based on Data Available at the Allen Mouse Brain Atlas and Allen Human Brain Atlas
Source: Neurol Res Int. 2017 May 23;2017:7138926. doi: 10.1155/2017/7138926 (PMC5463198; doi:10.1155/2017/7138926)

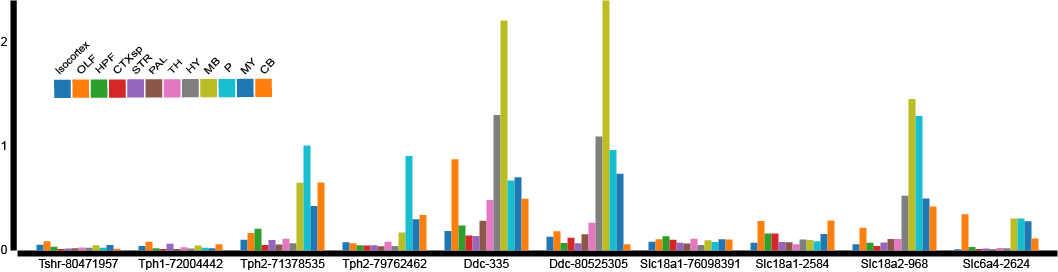

Supplement: Supplementary file 1 — Procedure details about databases, URLs and search. [file 7138926.f1.zip › Supplementary files/Fig S1. Supplementary.png]

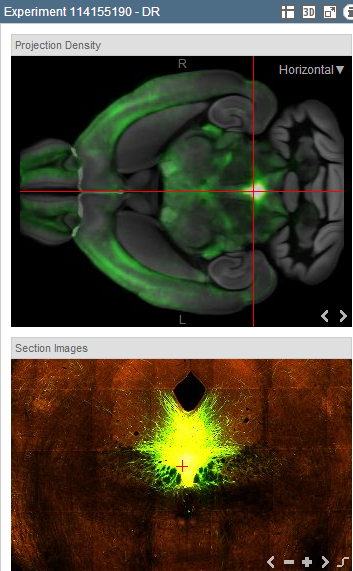

Supplement: Supplementary file 1 — Procedure details about databases, URLs and search. [file 7138926.f1.zip › Supplementary files/Fig S10. Suppl.png]

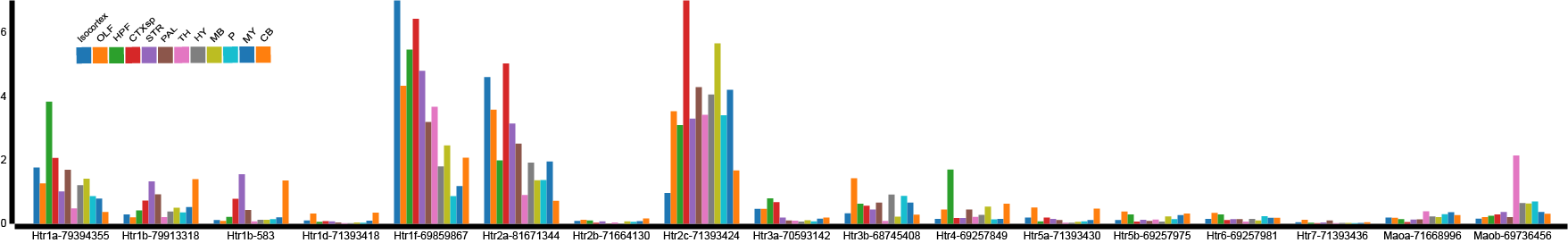

Supplement: Supplementary file 1 — Procedure details about databases, URLs and search. [file 7138926.f1.zip › Supplementary files/Fig S2. Supplementary.png]

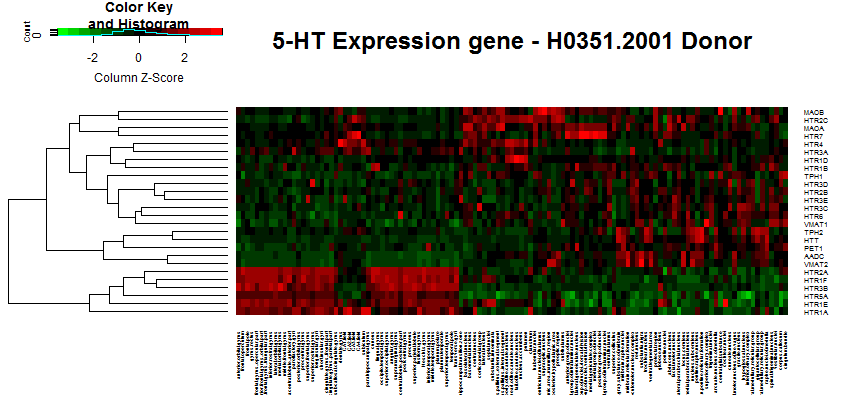

Supplement: Supplementary file 1 — Procedure details about databases, URLs and search. [file 7138926.f1.zip › Supplementary files/Fig S3 Suppl.png]

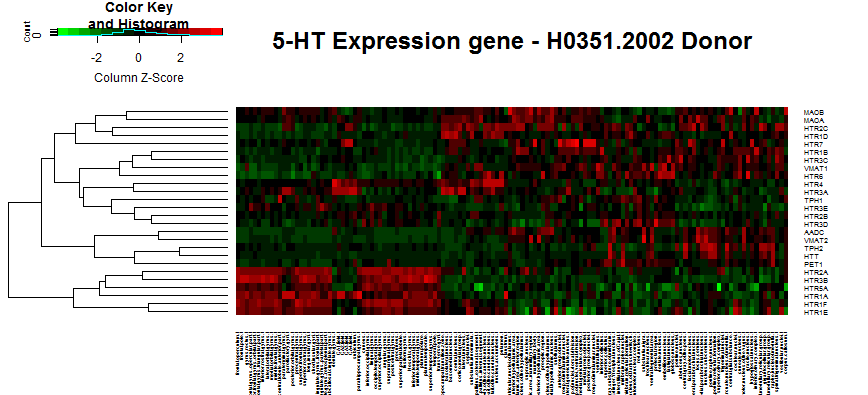

Supplement: Supplementary file 1 — Procedure details about databases, URLs and search. [file 7138926.f1.zip › Supplementary files/Fig S4 Suppl.png]

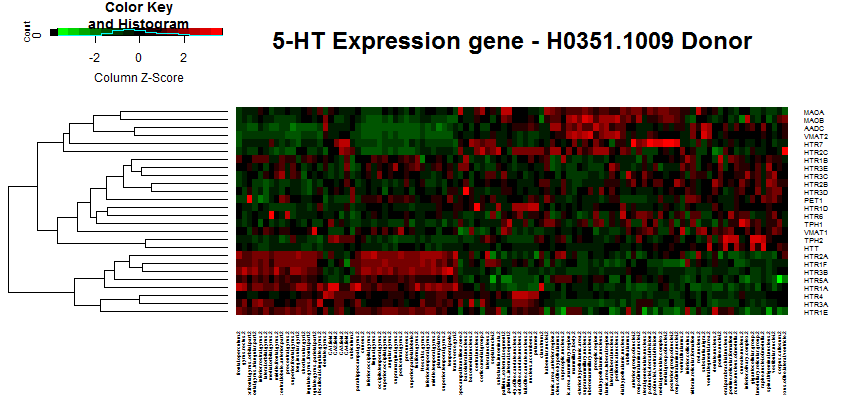

Supplement: Supplementary file 1 — Procedure details about databases, URLs and search. [file 7138926.f1.zip › Supplementary files/Fig S5 Suppl.png]

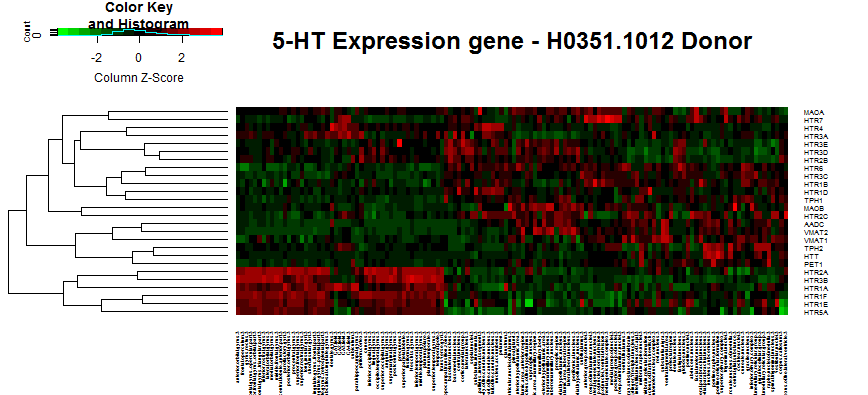

Supplement: Supplementary file 1 — Procedure details about databases, URLs and search. [file 7138926.f1.zip › Supplementary files/Fig S6 Suppl.png]

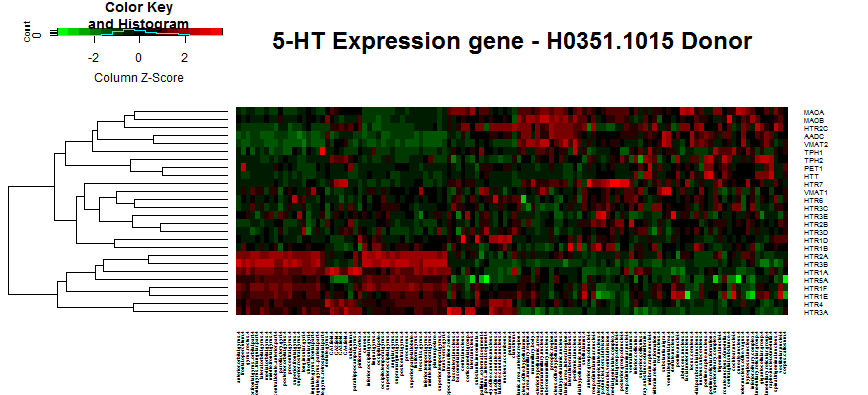

Supplement: Supplementary file 1 — Procedure details about databases, URLs and search. [file 7138926.f1.zip › Supplementary files/Fig S7 Suppl.png]

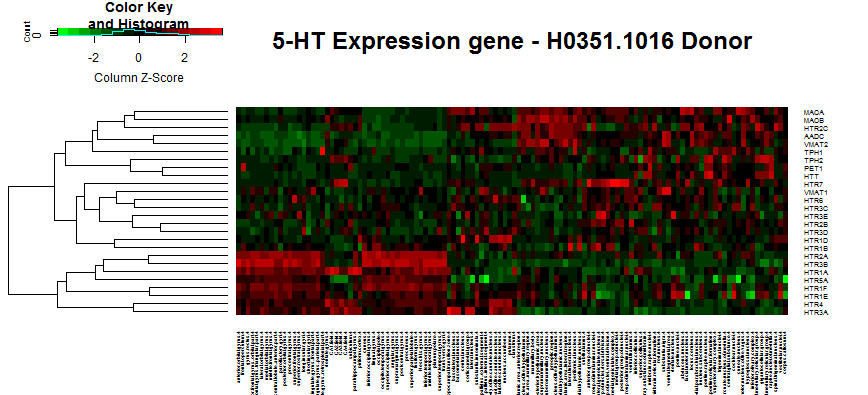

Supplement: Supplementary file 1 — Procedure details about databases, URLs and search. [file 7138926.f1.zip › Supplementary files/Fig S8 Suppl.png]

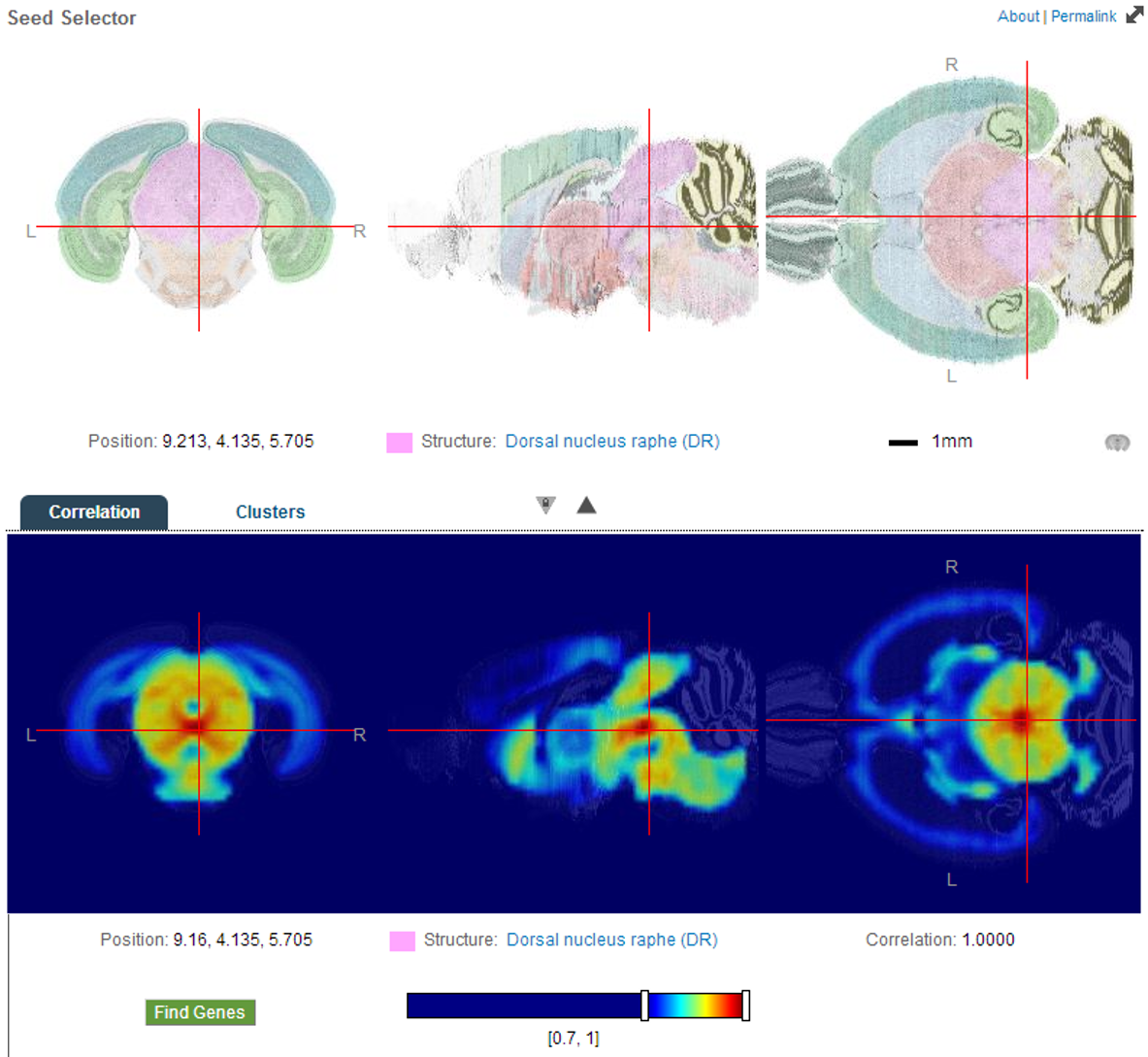

Supplement: Supplementary file 1 — Procedure details about databases, URLs and search. [file 7138926.f1.zip › Supplementary files/Fig S9 Suppl.png]
